# A Cluster Randomized-Controlled Trial of the Impact of the *Tools of the Mind* Curriculum on Self-Regulation in Canadian Preschoolers

**DOI:** 10.3389/fpsyg.2017.02366

**Published:** 2018-01-17

**Authors:** Tracy Solomon, Andre Plamondon, Arland O’Hara, Heather Finch, Geraldine Goco, Peter Chaban, Lorrie Huggins, Bruce Ferguson, Rosemary Tannock

**Affiliations:** ^1^Department of Psychiatry, The Hospital for Sick Children, Toronto, ON, Canada; ^2^Département des Fondements et Pratiques en Éducation, Université Laval, Quebec City, QC, Canada; ^3^Lawrence S. Bloomberg Faculty of Nursing, University of Toronto, Toronto, ON, Canada; ^4^School of Social and Community Services, George Brown College, Toronto, ON, Canada; ^5^Neurosciences and Mental Health, The Hospital for Sick Children, Toronto, ON, Canada; ^6^Institute of Medical Science, Faculty of Medicine, University of Toronto, Toronto, ON, Canada; ^7^YMCA of Greater Toronto, Toronto, ON, Canada; ^8^Department of Psychology, University of Toronto, Toronto, ON, Canada; ^9^Department of Psychiatry, University of Toronto, Toronto, ON, Canada; ^10^Applied Psychology and Human Development, Ontario Institute for Studies in Education, University of Toronto, Toronto, ON, Canada

**Keywords:** tools of the mind, self-regulation, executive function, preschool, curriculum, intervention

## Abstract

Early self-regulation predicts school readiness, academic success, and quality of life in adulthood. Its development in the preschool years is rapid and also malleable. Thus, preschool curricula that promote the development of self-regulation may help set children on a more positive developmental trajectory. We conducted a cluster-randomized controlled trial of the Tools of the Mind preschool curriculum, a program that targets self-regulation through imaginative play and self-regulatory language (Tools; clinical trials identifier NCT02462733). Previous research with Tools is limited, with mixed evidence of its effectiveness. Moreover, it is unclear whether it would benefit all preschoolers or primarily those with poorly developed cognitive capacities (e.g., language, executive function, attention). The study goals were to ascertain whether the Tools program leads to greater gains in self-regulation compared to Playing to Learn (YMCA PTL), another play based program that does not target self-regulation specifically, and whether the effects were moderated by children’s initial language and hyperactivity/inattention. Two hundred and sixty 3- to 4-year-olds attending 20 largely urban daycares were randomly assigned, at the site level, to receive either Tools or YMCA PTL (the business-as-usual curriculum) for 15 months. We assessed self-regulation at pre-, mid and post intervention, using two executive function tasks, and two questionnaires regarding behavior at home and at school, to capture development in cognitive as well as socio-emotional aspects of self-regulation. Fidelity data showed that only the teachers at the Tools sites implemented Tools, and did so with reasonable success. We found that children who received Tools made greater gains on a behavioral measure of executive function than their YMCA PTL peers, but the difference was significant only for those children whose parents rated them high in hyperactivity/inattention initially. The effect of Tools did not vary with children’s initial language skills. We suggest that, as both programs promote quality play and that the two groups fared similarly well overall, Tools and YMCA PTL may be effective curricula choices for a diverse preschool classroom. However, Tools may be advantageous in classrooms with children experiencing greater challenges with self-regulation, at no apparent cost to those less challenged in this regard.

**Clinical Trial Registration:** ClinicalTrials.gov, identifier NCT02462733.

## Introduction

We report the results of a cluster-randomized controlled trial of the effectiveness of a preschool curriculum aimed at improving children’s self-regulation. Self-regulation refers to the ability to exert control over one’s thoughts, feelings and behavior. It is involved in delaying gratification, sustaining focus in the midst of distraction and suppressing strong reaction in provocative situations, opting instead to apply reason. Language plays a central role in self-regulation. According to Vygotsky (1967, 1978), language is not only a cognitive tool for social communication but also permits control over one’s own cognitive processes such as memory and attention. Empirical evidence supports this proposition; toddler’s vocabulary predicts the development of self-regulation even after controlling for general cognitive development (Vallotton and Ayoub, 2011).

Self-regulation is related to the construct of executive function, neurocognitive processes that exert a top down influence on goal-directed behavior. Characterizations of the relation between self-regulation and executive function vary somewhat in the literature, but researchers generally agree that the core processes of executive function – working memory, inhibition and cognitive flexibility – are critical to self-control. Executive function refers to the cognitive aspect of self-control while self-regulation is concerned with behavior, including emotionally laden behavior, in the social context (see e.g., Blair and Ursache, 2011; Hofmann et al., 2012; Zelazo and Carlson, 2012). In the developmental literature, executive function is typically assessed with cognitive tasks administered to children individually, in a controlled setting, while self-regulation is captured through observation or by asking parents and teachers to complete questionnaires regarding children’s everyday behavior. Executive function has been linked to the pre-frontal cortex, which undergoes rapid development in the preschool years (Carlson, 2005; Zelazo and Carlson, 2012), although its development is susceptible to experiential and life stress factors. Several studies have shown that children from low socioeconomic status (SES) families consistently lag their higher SES peers in performance on executive function tasks (Noble et al., 2005, 2007).

Early challenges with self-regulation have considerable long-term consequences. A recent study following a cohort of children from birth to 32 years of age revealed that early childhood self-control predicted health and psychiatric problems, financial security and even criminality in adulthood, after controlling for intelligence and SES (Moffitt et al., 2011). These challenges are already apparent when children enter school, a critical transition that sets the stage for long-term learning. On a recent survey, more than half of a representative sample of American kindergarten teachers attributed children’s difficulties in kindergarten to challenges with following directions and maintaining attention. Indeed, teachers ranked these skills as more critical to early school success than content knowledge (Rimm-Kaufman et al., 2000). These observations are supported by findings that self-regulation is more strongly associated with school success than IQ or entry level reading and mathematics skills (Vitaro et al., 2005; Blair and Razza, 2007). Several studies have linked self-regulation to academic achievement, with better self-regulation related to better outcomes (Bull and Scerif, 2001; Welsh et al., 2010; Pingault et al., 2011).

Recent evidence suggests that self-regulation and executive function are both malleable, even in early childhood, and there is evidence that intervention might help to improve their development during this time (Diamond and Lee, 2011; Blakey and Carroll, 2015; Ling et al., 2016). Such findings are promising as Moffitt et al. (2011) have shown that children whose rank on measures of self-control improves between childhood and adolescence fare better in adulthood than their peers whose rank remains relatively stable. This could be because interventions that improve executive function early on might help to close academic achievement gaps down the road, with long term benefits for employment and overall wellbeing. Indeed, children with the weakest executive function skills, who tend also to struggle more academically, seem to gain the most from interventions that target self-regulation (Diamond and Lee, 2011; Blair and Raver, 2014). With a substantial percentage of 3- and 4- year olds now attending preschool (Kena et al., 2014) and evidence that preschool curricula can have a positive impact on school readiness (see, e.g., Gormley et al., 2005; Assel et al., 2007; Domitrovich et al., 2007), it seems reasonable to consider whether preschool curricula that targets the development of self-regulation might help set children on a more positive trajectory at the start of formal schooling.

### Tools of the Mind

One program that has garnered increasing attention in recent years for its potential to improve self-regulation is the Tools of the Mind curriculum (Tools; Bodrova and Leong, 2007). Tools is based on Luria’s (1966) and Vygotsky’s (1967, 1978) theories of cognitive development in which the social context of learning, imaginative play, language, and other cognitive tools play a critical role. Tools aims to improve self-regulation by providing frequent, structured opportunities for children to use these cognitive tools to practice self-regulation in the social context. The Tools daily routine is built around a set of activities carefully scaffolded by teachers that have a clear self-regulatory component. A substantial amount of time is devoted to pretend play. Children work with their teachers to choose a character (e.g., be a ‘doctor’), draw a play plan on paper and must then act in accordance with their plan, inhibiting the impulse to act out of character. Teachers refer children back to their play plan should they veer from their designated role. Children are taught to use a variety of cognitive tools, including language (to self and to others), to help regulate their behavior. For example, several activities require that children talk aloud as they complete the appropriate actions such as saying “clap” every time the task requires them to clap their hands. In other activities children are given pictorial cues (e.g., of a pair of ears or a mouth) to help them take turns listening and talking, and to self-regulate inappropriate behavior.

Tools is now used at numerous pre-primary sites in 20 States and in a few sites in Canada. Teachers in the entire country of Chile have been trained in Tools pedagogy (Farran and Wilson, 2014). Tools currently reaches over 30, 000 children^1^ and continues to receive a fair amount of public attention; in 2001, the United Nations Education, Scientific and Cultural Organization (UNESCO) added Tools to their list of exemplary instructional innovations^2^ and media coverage of the impact of Tools has appeared in major publications such as the New York Times and also on National Public Radio ^3^^,^^4^. Yet efforts systematically to evaluate the effectiveness of Tools for improving self-regulation, socio-emotional and academic outcomes are limited, and the results have been mixed. A summary of this work is shown in **Table 1**.

**Table 1:** Summary of previous research on the effectiveness of the Tools preschool curriculum.

Reference	Programs,	Age, SES	Duration of	Fidelity	Key findings
	sample size		exposure	measured	
**(A) Published studies.**					
Blair and Raver, 2014	(1) Tools - K *n* = 443 (2) BAU (state curriculum) *n* = 316	K Mixed SES	∼4–5 months	No	Tools group had significantly greater stress reduction, better improvement in working memory and processing speed, were faster but not more accurate on executive function, had greater gains in math, reading, non-verbal reasoning and vocabulary, but effects generally stronger in high poverty schools (EF’s range 0.08–0.14. overall and 0.28–0.82 in high poverty schools).
Diamond et al., 2007	(1) Tools - P *n* = 85 (2) DBL *n* = 62	4–5 years Low SES	1 or 2 school years (2 cohorts)	Yes	Tool group scored significantly better on executive function tasks and academic achievement at post, but no baseline data and achievement data available for Tools children only.
Barnett et al., 2008	(1) Tools - P *n* = 88 (2) DBL *n* = 122	3–4 years Low SES	∼6 months	Yes	Teachers rated Tools group significantly lower on problem behavior at post, but no baseline data collected.
**(B) Unpublished studies.**					
Farran and Wilson, 2014^∗^	(1) Tools - P *n* = 499 (2) BAU (variable, modal was CC) *n* = 379	4.5 years at pre-test Low SES	∼1 school year	Yes	No significant benefits to Tools group. BAU group improved significantly more on measures of early reading, math, working memory in Kindergarten and spelling, attention and self-regulation in 1st grade.
Lonigan and Phillips, 2012	(1) Tools - P (2) LEPCP (3) LEPCP + pretend play from Tools - P (4) BAU (typically a version of HS or CC) *n* = 2, 564 altogether	2.5–6 years at pre-test SES not given	∼1 school year	No	No significant benefits to Tools group or to LEPCP plus pretend play from Tools group. No overall advantage to any group on self-regulation. Tools group scored significantly lower than BAU group on reading, and than LECPC group on reading and vocabulary, at post.
Clements et al., 2012	(1) BB (2) BB + Tools - P self-regulation component (3) Control *n* = 826 altogether	4 year-olds SES not given	∼1 school year	No	No significant benefits to adding the self-regulation component of Tools – P to BB. No significant differences between any groups on the outcome measures.
Morris et al., 2014	(1) HS (2) HS+ PATHS (3) HS+ pretend play from Tools - P (4) HS + IY *n* = 2763 altogether	Pre-K Low SES	∼1 school year	Yes	Compared to HS only (the control group), the HS plus pretend play component from Tools group had significantly better emotion knowledge but it did not translate to better problem-solving.

*Tools, Tools of the Mind; -K, Kindergarten; -P, preschool; BAU, Business as Usual; SES, socioeconomic status; EF, effect size; DBL, District Balanced Literacy; LEPCP, Literacy Express Comprehensive Preschool Curriculum; HS, High Scope; CC, Creative Curriculum; BB, Building Blocks; IY, Incredible Years. ^∗^See also Wilson and Farran (2012) and Farran et al. (2013).*

Seven studies have evaluated the impact of Tools of which three – those with positive effects – have been published (see **Table 1**). Only one study evaluated the kindergarten version; comparing children who received Tools instruction to those who received the business as usual instruction (BAU, the state curriculum) in Kindergarten. Children were assessed in the fall and spring of kindergarten and the fall of first grade. The authors found significant benefits to the Tools group including greater stress reduction, and greater improvement on cognitive and academic measures, with some effects carrying over to first grade. Of note, the effect sizes in the overall sample were relatively small compared to those in high poverty schools (see **Table 1**; Blair and Raver, 2014).

Two published studies investigated the impact of the preschool version of Tools (Diamond et al., 2007; Barnett et al., 2008). Both studies compared low SES children who received Tools to those who received a literacy-focused curriculum. Diamond et al. (2007) found that children in the Tools group performed significantly better than their non-Tools peers on executive function measures, and that the more demanding the executive function task the more strongly performance was correlated with measures of academic achievement. Barnett et al. (2008) reported that Tools significantly improved classroom quality, but the only student measure with a significant finding (from analyses taking the hierarchical nature of the data into account) was that teachers rated Tools children significantly lower on a brief problem behavior scale (measuring externalizing) compared to their non-Tools peers. However, as no baseline data were collected in either study and achievement data were only available for the Tools group in the Diamond et al. study, it remains unclear whether the apparent benefits to the Tools groups reflect an improvement in functioning and if those benefits were unique to the children who received Tools instruction.

Two unpublished studies investigating the Tools preschool program were reported at the meeting of the Society for Research on Educational Effectiveness in 2012 and 2013. These studies involved large samples and rigorous methodology, including assessments at pre and post. Farran and Wilson (2014; see also Wilson and Farran, 2012; Farran et al., 2013) compared children who received the Tools curriculum to those who received the BAU curriculum, which varied across participating sites. Lonigan and Phillips (2012) compared the effectiveness of four curricula; a skills-focused curriculum, the Tools curriculum, the skills focused curriculum enhanced by the pretend play component of the Tools curriculum, and the BAU curriculum that varied across participating sites. The results showed no significant benefits to children who received either the Tools program or the skills focused curriculum enhanced by the pretend play component of Tools. Moreover, both studies found greater advantages to the comparison group children on a range of academic and self-regulation or executive function outcomes (see **Table 1**).

Two additional unpublished studies investigated the impact of adding only the pretend play component of Tools to existing curricula. Clements et al. (2012) compared 4-year-old children who received Building Blocks (BB, a math focused curriculum), to children who received BB plus the pretend play component of Tools, to a control group of children and Morris et al. (2014) investigated the impact of three enhancements to the curricula in preschools in the Head Start program; The Incredible Years (which focuses on teachers ability to create an organized, positive classroom context), Preschool PATHS (which provides teachers with a set weekly lessons focused on improving emotion knowledge and problem-solving skills) and the pretend play component of Tools. Neither study found any significant advantages to including the pretend play element of Tools to the existing curricula. Morris et al. (2014) reported a slightly greater gain in emotion knowledge in the Tools group compared to the group with no enhancements but it did not translate to better problem-solving skills. In sum then, previous research on the effectiveness of Tools has produced some positive evidence for Kindergarten and limited, mixed evidence for the preschool version of the program.

It is difficult to know what to make of the inconsistent pattern of findings in the research to date in part because previous studies have varied considerably in methodology, including sample size and characteristics (such as age and SES), the duration of Tools instruction, and whether children received the Tools program or their regular instruction enhanced by aspects of Tools (see **Table 1**). The research to date has also focused on different key outcomes (measuring only executive function, only self-regulation or both) as well as in the actual measures used to assess them. However, two commonalities amongst previous studies are worthy of closer consideration for their potential to provide further insights regarding why Tools might sometimes be effective. The first commonality is that the impact of Tools has typically been compared to that of curricula focused on academic skills, especially literacy (see e.g., Diamond et al., 2007; Barnett et al., 2008; Blair and Raver, 2014). Hence, it is not clear whether the apparent benefits of Tools might be due to the program’s focus on self-regulation or on improving quality play. The second commonality is that positive effects of Tools tended to occur in low SES samples (Diamond et al., 2007; Barnett et al., 2008; Blair and Raver, 2014). Low SES has been linked to greater challenges on a number of measures that are critical for school readiness including language, executive function and hyperactivity/inattention (Noble et al., 2005; Fitzpatrick et al., 2014; Foulon et al., 2015). Thus, it could be that Tools is most effective in children for whom these abilities are relatively undeveloped. To be sure, not all studies with low SES samples have found positive evidence of Tools (e.g., Farran and Wilson, 2014; Morris et al., 2014). However, as previous research has varied widely in how children were assessed, including their cognitive abilities, it is possible that positive effects occurred in samples with especially poor cognitive skills or for whom low cognitive performance might have been more homogeneous (e.g., Diamond et al., 2007; Barnett et al., 2008). We explored these ideas in the present study.

### Rationale for the Present Study

The goals of the present study were to ascertain whether the Tools program, which targets self-regulation through imaginative play and self-regulatory language, leads to greater gains in self-regulation compared to Playing to Learn (YMCA PTL), another play based program that does not target self-regulation specifically, and whether the effects were moderated by children’s initial language and hyperactivity/inattention. As both programs are play based, and devote considerable time in their daily routine to improving quality play, but only Tools explicitly targets the development of self-regulation (see the section “Materials and Methods” for program descriptions), the study offered the opportunity to explore whether or not any gains resulting from Tools was related to the program focus on self-regulation over and above its focus on improving quality play.

It also afforded the opportunity to investigate whether previous findings of positive effects of Tools in low SES children is related to their relatively less developed cognitive skills.^5^ At issue was whether children with low language and high hyperactivity/inattention might gain more from Tools instruction. It is possible that the Tools program emphasis on language may boost language skills in children with low language and thus improve their capacity to use language to regulate their behavior with self-directed speech and also to communicate more effectively with others. Children with high hyperactivity/inattention may benefit from having more frequent opportunities to practice self-regulation integrated into their daily routine because at least some opportunities to do so that arise as par for the course of most preschool curricula (e.g., taking turns with a coveted toy or activity) may be lost on them. Accordingly, we analyzed the data to determine if the effectiveness of Tools varied as a function of children’s initial levels of language and hyperactivity/inattention.

We included well-established measures of executive function and also of self-regulation to paint a clearer picture of the impact of the Tools curriculum and to facilitate comparisons to previous studies. To better understand the nature of the impact of Tools on executive function, we included two measures of executive function with similar cognitive demands but different response modalities, one behavioral and one verbal.

Finally, it is also worth mentioning that the study was of considerable practical import. The opportunity to work with preschoolers in the target age range attending a network of childcare sites was particularly appealing at the time because it occurred in the last year before the final school year rollout of a free, full-day kindergarten program (FDK), which would soon be available to all 4-year-old children through the public school system. Interest in the study was intensified by ongoing discussion regarding the type of curriculum that might be best suited for the new program.

## Materials and Methods

### Study Overview

The study was a registered cluster-randomized controlled trial, clinical trials.gov identifier NCT02462733, carried out in multiple childcare centers run by the YMCA Canada – a charitable, community-based organization – in a large urban center, in Ontario, Canada. A total of 20 sites, each with one participating class, were randomly assigned to teach either the Tools of the Mind (TOOLS, *n* = 10 classes; 109 preschoolers) or the YMCA Playing to Learn (YMCA PTL, *n* = 10 classes; 86 preschoolers) curriculum. The different number of participating preschoolers in the two groups reflects the variation in the number of children in the target age range attending the participating sites, prior to random assignment of sites to curricula. The YMCA PTL preschool curriculum was the business-as-usual curriculum in use throughout the YMCA prior to the study. Each class was led by an early childhood educator (ECE), with an assistant teacher. Teachers received training in their respective curricula and used only the method of instruction assigned to their site for about 15 months; from March 2012 to June of 2013, inclusive. We assessed children at three time points; at start of the study (T1), around 8 months later (T2) and at the end of the study (T3)^6^. We also assessed the teacher’s fidelity of implementation of the Tools program at three time points; at 7, 11, and 14 months of implementation (F1, F2, and F3, respectively). There were two cohorts of participating children: Cohort A who entered the study at T1 and Cohort B who entered the study at T2 (see the section on “Participants” for further details).

Our primary outcome measures comprised two executive function tasks as well as parent and teacher reports of children’s behavior at home and school, The two measures of executive function were the Day/Night task (D/N; Gerstadt et al., 1994) and the Head-To-Toes version of the Head-Shoulders-Knees-Toes task (HTT; Ponitz et al., 2009). The questionnaires were the parent and teacher versions of the Strengths and Difficulties Questionnaire (SDQ-P, SDQ-T, respectively; Goodman, 1997, 1999) and the Social Competence and Behavior Evaluation Scale, which was completed by teachers only (SCBE-30; LaFreniere and Dumas, 1995). We used the total difficulties score from the SDQ-P and SDQ-T (see measures) as outcome measures. For the analyses looking at the effect of Tools as a function of hyperactivity/inattention we used the hyperactivity/inattention subscale from the SDQ-P at baseline reasoning that parent reports are unbiased compared to teachers who also delivered the curriculum. For the analyses looking at the effect of Tools as a function of initial language, we used the Peabody Picture Vocabulary Test (PPVT-4, Dunn and Dunn, 1997).

The data were collected as part of a larger investigation of the development of self-regulation that included a number of additional measures. We used these measures in the present study to help characterize the groups at baseline. The additional measures tapped children’s expressive language (Expressive Vocabulary Test, EVT-4, Williams, 2007), as well as their early reading and math skills (Get Ready To Read, GRTR, Whitehurst and Lonigan, 2001; Point-to-X, PTX, Wynn, 1992). Teachers also completed a questionnaire on children’s overall development (the Early Development Index, EDI, Janus and Offord, 2007).

### Ethics Statement

The study was approved by the research ethics board at the Hospital for Sick Children in Toronto, Canada and also by the YMCA Canada organization. Teachers provided written informed consent and parents provided written informed consent for their participating children. Consent for teachers and for children in cohort A was obtained prior to random assignment of sites to conditions. Consent for children in cohort B was obtained after the randomly assigned curriculum was already in place.

### Participants

The participating sample was drawn from a network of childcare sites operated by YMCA Canada. Resources as well as teacher credentials and professional development were therefore standardized across sites at the study outset. We targeted all of the sites in the network located in areas where local elementary schools were not scheduled to offer the FDK program until after the study period. The 20 eligible sites spanned the city limits and served populations that were ethnically and socio-economically diverse.^7^ The mean percentage of students in the preschool program receiving a fee subsidy was 54% (range 19–100) and 59% (range 1–100) in the Tools and YMCA PTL sites, respectively. Note that as fee subsidy data were not available for individual children, we were unable to use this proxy for SES in our analyses. All of the site directors agreed to participate in the study.

Teachers were recruited to participate in the study if they were accredited Early Childhood Education teachers and were not expecting to take a leave of absence during the study period. Their participation was voluntary.

Children were recruited to participate if they were 3 or 4 years of age, had sufficient grasp of English, and did not have any developmental challenges serious enough to preclude full participation in the curriculum, as judged by their teacher. They were expected to remain at the daycare for the study duration. The participating children at each site were grouped together into a single mixed-age classroom along with other non-participating peers who did not meet the eligibility criteria for participation in the study or whose parents did not return a signed consent form in time for baseline data collection.

**FIGURE 1:**
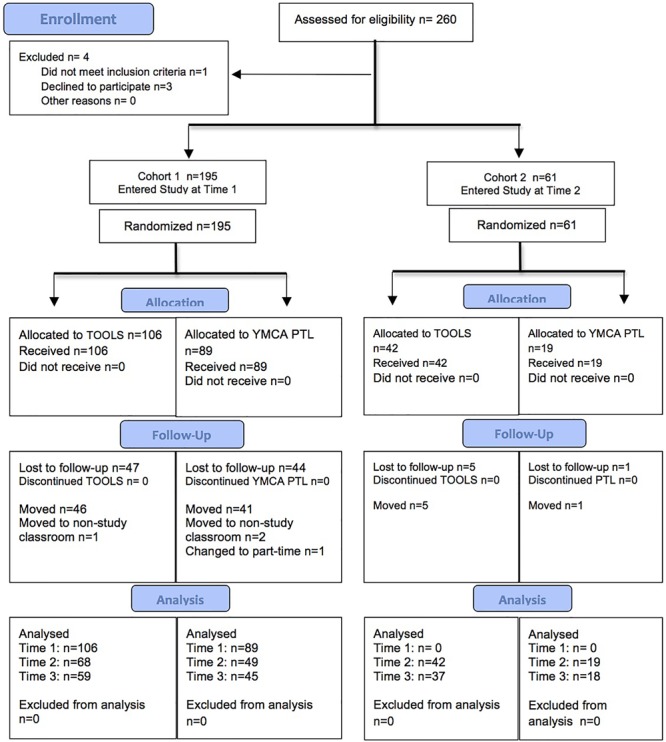
Consort flow diagram of study participants.

Cohort A: **Figure 1** shows the Consolidated Standard of Reporting Trials Organization (CONSORT) diagram of participant flow through the study. The details for Cohort A are shown on the left side of the figure. We received signed consent for 199 children at T1. Three children refused to participate and 1 child who participated exhibited developmental challenges that prohibited a fair administration of the battery of measures. These 4 children were dropped from the study, leaving 195 participants in Cohort A at T1. There were 106 children in the TOOLS group (58 boys and 48 girls, mean age = 45.1 months, range 37.2–55.34 months) and 89 children in the YMCA PTL group (47 boys and 42 girls, mean age = 45.9 months, range 36.5–62.3 months). The two groups did not differ in mean age at the start of the study.

**Table 2** shows baseline performance on the study measures for cohort A at T1, by curriculum. Independent samples t-tests indicated that the groups did not differ significantly on either the D/N, HTT, or on the total difficulties score on either the SDQ-P or SDQ-T. The only significant difference between the groups was that teachers rated Tools children significantly higher than their YMCA PTL peers on the anxiety-withdrawal scale on the SCBE-30, but the difference between the group means was small (difference = 0.38, *p* < 0.0001). There were no significant differences between the groups on any of the additional measures. All tests were based on the Bonferroni adjusted *p*-level for multiple comparisons which was *p* < 0.02.

**Table 2:** Performance at study entry for cohort A (T1) and cohort B (T2).

	Cohort A		Cohort B	
	YMCA PTL (*n* = 89)	Tools (*n* = 106)	YMCA PTL (*n* = 19)	Tools (*n* = 42)
	Mean (*SD*)	Mean (*SD*)	Mean (*SD*)	Mean (*SD*)
**Primary measures**				
Day/night	9.87 (4.7)	10.88 (4.8)	11.8 (4.3)	12.71 (4.1)
Head to toes, score out of 20	5.99 (7.6)	6.60 (7.1)	6.47 (7.4)	5.72 (6.86)
Head to toes, score out of 10	3.60 (4.2)	3.94 (3.9)	3.53 (4.1)	3.42 (3.8)
SDQ-parent total difficulties	8.95 (5.6)	8.72 (5.7)	8.44 (5.7)	7.73 (5.2)
SDQ-teacher total difficulties	6.54 (5.5)	7.76 (5.7)	6.56 (5.5)	9.38 (5.1)
SCBE-30 anger/aggression	2.06 (0.67)	2.23 (0.85)	1.85 (0.62)	2.13 (0.51)
SCBE-30 anxiety/withdrawal	1.87 (0.58)*	2.25 (0.67)	2.18 (0.71)	2.23 (0.80)
SCBE-30 social competence	2.81 (0.83)	2.82 (0.88)	3.00 (0.90)	2.95 (0.75)
PPVT-4 standard score	101.5 (15.0)	100.74 (15.2)	98.35 (18.4)	109.85 (12.00)
**Additional measures**				
EVT-4 standard score	106.4 (16.5)	105.8 (15.5)	99.74 (20.2)	112 (14.6)
GRTR (reading)	12.59 (5.1)	12.02 (4.4)	12.79 (4.49)	13.73 (4.52)
Point-to-X (math)	11.33 (2.8)	10.97 (2.3)	12.41 (3.5)	10.95 (2.9)
EDI_R physical well-being	9.11 (0.95)	8.73 (1.1)	9.08 (0.95)	8.32 (1.0)
Social competence	7.76 (1.8)	7.20 (1.9)	7.44 (2.1)	6.51 (1.77)
Emotional maturity	7.7 (1.4)	7.2 (1.5)	7.5 (1.3)	6.85 (1.4)
Language and cognitive development	6.08 (2.1)	5.59 (1.9)	6.61 (1.9)	5.16 (1.9)
Communication and general knowledge	7.8 (2.2)	7.44 (2.5)	6.8 (2.1)	6.24 (2.6)

*SDQ, Strengths and Difficulties Questionnaire; SCBE-30, Social Competence and Behavior Evaluation Scale; PPVT-4, Peabody Picture Vocabulary Test, 4th ed.; EVT, Expressive Vocabulary Test 4th ed.; GRTR, Get Ready to Read; EDI, Early Development Inventory. We compared the group means within each cohort for each measure. *P* < 0.02 is the critical alpha level after Bonferroni adjustment for multiple comparisons. ^∗^denotes a significant difference between the two groups in cohort A, based on the adjusted *p*-level.*

It is important to note here, that there was considerable attrition in the summer of 2012 (about 5 months after the start of the study), due to unforeseen circumstances. Seventy-eight children in Cohort A (Tools; 23 boys and 15 girls, YMCA PTL; 21 boys and 19 girls) left the study before T2. Three children moved to a different classroom in the same daycare, 1 child changed to part-time attendance and 2 children withdrew from the study (1 from each condition), but the remaining 72 children left the daycare altogether. It is unlikely that the attrition was related to either curricula, since attrition rates were comparable in the two conditions. It is also unlikely that the relatively large number of children who left the study can be fully accounted for by the typical reasons for attrition, such as a change of residence or parental leave to care for a new sibling. Rather, we believe that it was largely due to an unanticipated effect of the rollout of FDK. Although the attrition was distributed across sites, some sites were affected more than others, namely those located in the city core and at suburban transportation hubs that may have served commuter populations. Children may have attended these sites because they were close or en route to a parent’s place of employment but resided in neighborhoods where local schools were scheduled to introduce FDK in the fall of 2012. There may therefore have been withdrawn to take advantage of the free program. This notion is supported by the timing of the attrition, the fact that most of the children left the daycare altogether (as opposed to switching to a non-study room), and because the daycare staff suggested that FDK was the most likely reason for leaving.

To determine the impact on the study of the attrition before T2, we compared children who left the study to those who stayed on the primary measures and also on the additional measures administered at baseline. Independent-samples *t*-tests revealed that leavers and stayers did not differ significantly in chronological age (mean age was 45.3 and 44.6 months, respectively, at the start of the study). Furthermore, although leavers generally scored somewhat less well than stayers, the differences between group means did not reach statistical significance.^8^ A further 11 children in Cohort A (5 TOOLS, 6 YMCA PTL) left the daycare before T3; 1 moved to another classroom and 10 left the daycare altogether, due either to moving residence or to parental leave (based on teacher reports).

Cohort B: Given the considerable investment of resources, the daycare staff’s enthusiasm to continue on with the study, and in order to improve statistical power for the data analysis, we recruited an additional cohort of 3- and 4-year olds (Cohort B) into the study at T2, from the participating sites. Details regarding the flow of participants in Cohort B are shown on the right side of **Figure 1**. Cohort B comprised 61 children; 42 children in the TOOLS group (25 boys and 17 girls, mean age = 42.4 months, range 37.0–50.0 months) and 19 children in the YMCA PTL group (8 boys and 11 girls, mean age = 43.5 months, range = 37.0–57.1 months). These children were already attending the participating sites but were not in the study classrooms at the time of recruiting. They became eligible for the study largely because they had achieved the minimum age criteria by T2. This meant that they were significantly younger than the children in cohort A at T2 (*p* < 0.0001; mean ages were 54.3 and 42.7 months, for cohorts A and B, respectively). Comparisons of the cohort A and cohort B children at T2 confirmed that, in general, cohort A was also developmentally more mature.^9^ After obtaining parental consent, cohort B children joined their cohort A peers in the participating classroom in their daycare. Hence, they received the curriculum that was randomly assigned to the classroom at the start of the study and already in place at T2. Independent-samples *t*-tests revealed no significant differences on any of the study measures between the Tools and YMCA PTL children who entered the study at T2 (see **Table 2**). Six children in cohort B (5 TOOLS, 1 YMCA PTL) left the daycare before T3, again due to moving or parental leave (based on teacher reports).

### Materials and Procedures

#### Program Descriptions, Teacher Training, Tools Fidelity

##### Program descriptions

Tools of the Mind (Tools; Bodrova and Leong, 2007) is a play-based, preschool and kindergarten curriculum that emphasizes self-control, language and literacy skills. The present study involved the preschool version of the program. Tools is based on Vygotsky’s (1967, 1978) social-cultural theory of child development in which development occurs in the context of the interactions between children and their social environment. These include interactions with peers as well as adults. Play, especially pretend play, is considered essential to propelling development. In pretend play, children adopt various social roles and implicitly agree to act in accordance with those roles, inhibiting the propensity to act out of character. Language is considered a critical tool for the formation of thought. Indeed, children employ a variety of tools to support their thinking. Initially, these tools are external such as a picture or language spoken aloud, but in time they become automatized and internalized as when children remember the significance of a picture or engage in internal self-talk to help regulate their own behavior.

The Tools curriculum comprises a set of explicit, scripted, teacher-directed activities that embody these ideas and that are aimed specifically at improving self-control. A considerable part of every day is devoted to pretend play, which begins with teachers helping children to formulate a play plan drawn on paper. Children are asked to think about the setting, the key roles and who will play them, the language their character might use, as well as the main events that will take place. They then draw – to the best of their ability – a depiction of the scenario. They are also encouraged to make marks, or draw letter-like forms, write letters, words or simple phrases to accompany their drawings, as appropriate for their skill level. During the pretend play sessions, teachers help children to self-monitor by reminding them of, or referring to the actual plan as needed, and suggesting additional activities and language for their character. Children also assist each other by pointing out and redirecting peers when they begin to act out of role.

Teachers provide additional support by integrating pretend play into other activities in the curriculum. For example, when the whole class is gathered on the carpet the teacher may adopt the role of a baker, model the actions of cutting up and distributing a pretend pizza, using relevant vocabulary. Teachers also stimulate children’s thinking about how various objects can be appropriated for use in the pretend scenario such as using a rectangular piece of cardboard as a telephone. Children are encouraged to practice the action sequences modeled by the teacher and to integrate them into their pretend play scenarios.

Language is also afforded a central role. It is the primary mechanism for introducing and for participating in the Tools activities. Children employ substantial overt, and eventually covert, speech to guide their actions on a variety of tasks such as when practicing writing. Teachers also help to build children’s vocabularies by identifying new words in books selected for classroom reading. During large group time, teachers introduce and model the use of new words, relevant to the theme of the children’s pretend play. Throughout the day, a variety of activities require children to articulate their ideas and to begin to make symbolic marks, construct letters, words and then simple phrases to express those thoughts. Hence, in addition to self-direct speech, there is a great deal of verbal exchange between students as well as between the students and the teacher in the Tools classroom.

Opportunities to practice self-regulation are also incorporated into both large and fine motor activities. For example, in the “freeze game,” the teacher plays rhythmic music while holding up a card depicting a stick figure in a particular stance. Children dance and when the music stops they must strike the pose in the picture. When the music recommences they begin dancing again and the sequence is repeated with a new card showing a different physical stance. In “pattern movement,” children are shown different shapes (e.g., triangle and square) and taught to execute a different movement for each shape (e.g., touch your chin for the triangle, clap for the square). The teacher then reveals a sequence of shapes, one shape at a time (e.g., square, triangle, and triangle), and children must perform the sequence of actions that corresponds with the shape pattern (e.g., clap, touch chin, and clap). Children are encouraged to label their actions aloud as they execute them.

Tools academic activities also have a clear self-regulation component. For example, in “buddy reading” children read aloud in pairs, with each child taking a turn as the reader or the listener. They are given pictures (of a mouth or an ear) to help them stay in their role. The listener is encouraged to ask the reader a question about the text when the reader has finished reading. The children then switch roles along with their accompanying pictures. Similarly, for “making collections” children are designated as either the counter or the checker and given pictures (of a hand or a checkmark) to help them stay in role. The counter’s role is to place the number of counters into a cup that matches the number of items shown on a “key card.” The checker checks and provides feedback so that the counter can make corrections. After several efforts with different quantities of counters, the children switch roles and pictures. In both tasks, the different roles become internalized over time and children no longer require the external symbols to support appropriate behavior.

Detailed manuals of the Tools program have been developed for use in in-service training, which consists of an admixture of workshops and in-class coaching, and also for teachers to use as an ongoing resource throughout the training period^10^.

Playing to Learn (YMCA PTL; Eden and Huggins, 2001; Martin and Huggins, 2015) is also a play-based preschool curriculum. A critical difference between Tools and YMCA PTL is that, whereas Tools is a more teacher-directed, prescribed approach, YMCA PTL is a child-centered, emergent curriculum. The teacher’s primary roles are to establish a safe, secure, social environment and to facilitate learning through play, following the child’s interest. The set up of the physical environment is seen as essential to encouraging quality play (but not necessarily pretend-play). YMCA PTL classrooms resemble home-like environments. A wide variety of materials are available to encourage play that supports children’s social, emotional and academic learning. Children who become disengaged may be enticed by different aspects of the environment to re-engage in play.

Teachers keep a flexible daily routine to encourage sustained, uninterrupted periods of play. Play is open-ended, creative and flexible, adapting to children’s needs, interests, and ideas as they change. Teachers act as play partners, enthusiastically entering the play scenario but only on the children’s invitation. They may modify or add to the experience and help to extend play according to level of interest, but the children continue to guide the play content.

Teachers are trained to observe children’s play, to reflect on, and to carefully document their interests. They are encouraged to capitalize on learning opportunities as they arise. For example, teachers may encourage children who are using blocks to build a fort to think about how the size and arrangement of the blocks influences its final structure, to count the number of blocks involved, the number of children the structure can accommodate as well as the structure’s affordances. Practicing self-control is an emergent property of these child-initiated activities. For example, teachers may help children to solve the problem of too few blocks for all of the children interested in the block building activity, by organizing themselves into teams and taking turns.

Teachers may also plan for innovative play opportunities but they are rooted in observations of the children’s interests. Moreover, play planning is flexible, can be adapted or even abandoned according to children’s changing interests and needs. For example, a teacher who observes some children’s growing interest in dinosaurs may set out a box containing various dinosaur paraphernalia such as miniature figures, dinosaur eggs, books and so on for children’s arrival the next day. She may set up outdoor play so that children can engage in digging for dinosaur “bones” (Martin and Huggins, 2015). The following day, the teacher will partner with the children on these dinosaur activities if they show an interest, but if children redirect their interests – such as spontaneously pretending to be riding on a bus – the teacher is flexible to enough to abandon the dinosaur idea and to apply efforts to the new scenario.

Opportunities for social, emotional and academic learning are embedded in play. For example, teachers encourage co-operation to solve problems, they model empathy, they may introduce new vocabulary in developing play on a particular topic, incorporate number and creative problem-solving into play such as in the fort building activity described above. Similarly, opportunities to practice self-regulation in play might occur when a teacher suggests turn-taking as a solution to sharing a highly desirable object or helps children to generate alternative solutions such as stopping to think about a conflict and using words instead of impulsive actions to express their dissatisfaction. Children are not routinely required to represent their play ideas visually, via drawing, symbolic mark-making or letter construction but drawing materials would be made available if they demonstrated an interest.

As with Tools, a YMCA PTL manual has been developed to guide classroom practice. In service training consists of professional development sessions and in-class coaching.

##### Teacher training

Prior to the study, all of the teachers, who were all ECE accredited, were fully trained in implementing the YMCA PTL curriculum. Upon joining the YMCA, they received an orientation to PTL followed by 4 further training sessions within the first 6 months and then 2 additional sessions in each subsequent year with the YMCA. Teachers were assessed for implementation fidelity by the participating organization, as part of an annual, general site evaluation. Regional supervisory staff provided ongoing coaching to help sustain implementation fidelity to organizational standards. For the YMCA PTL teachers only, YMCA PTL training, coaching support and evaluation continued as usual while the study was underway.

For teachers in the Tools classrooms, the YMCA PTL booster training, support and evaluation was suspended for the study duration. Instead, teachers received training in the Tools preschool curriculum by professional trainers from the Tools organization. Training was delivered incrementally, in five sessions, roughly evenly distributed throughout the study period. Teachers were trained in the core Tools activities (those essential to program implementation) at the beginning of the study and while data collection at T1 was underway, and the last session occurred about 2–3 months before data collection at T3. In between sessions, teachers continued to implement the core Tools program integrating any additional skills acquired at the most recent training. Two coaches, who received the same training as the teachers, as well as additional coaching training, provided ongoing support throughout the study. Each site received equal amounts of support from the two coaches during the study period. The Tools trainers also visited the participating sites following each training session and made recommendations to help support implementation fidelity.

##### Tools fidelity

Following similar published studies (see e.g., Diamond et al., 2007; Barnett et al., 2008), we focused on the implementation fidelity of Tools. Our primary aim was to establish that the Tools curriculum was in use in the Tools classrooms and not in the YMCA PTL classrooms. As reported above, ongoing coaching in YMCA PTL classrooms helped to ensure fidelity of implementation of the YMCA PTL curriculum to standards acceptable to the YMCA organization.

To capture fidelity of implementation of the Tools program, members of our research team completed the Tools Implementation Checklist (TIC) we created specifically for the present study. The TIC comprised a list of the Tools activities that would be expected to take place in the classroom based on teacher training. Each activity was broken into its essential elements laid out in the Tools manual and observers checked whether or not they observed each element. The 21 core activities teachers were expected to implement throughout the study (following the first training session) comprised 119 observable elements. Five additional activities were added at F2 and 1 further activity was added at F3 as teachers progressed in their training and the students in their learning. These additional activities comprised 35 observable elements at F2 and an additional 6 observable elements at F3. Hence, the total number of possible observable elements or items on the TIC was 119, 154, and 160 at F1, F2, and F3, respectively.

We assessed fidelity of implementation of the Tools program at all 20 participating sites, at three time points (as explained above). At each time point, a pair of observers attended each site for a full day and completed the TIC. The observers were graduate students in a combined early childhood education and elementary/junior teacher accreditation program nearing the end of their studies. A different pair of observers completed the observations at each time point. The observers were blind to the study hypotheses and to the assignment of sites to the two curricula. They attended the same site, on the same day, but completed their own copy of the TIC, independently without conferring. We report inter-rater reliability and percent implementation of the Tools activities, at the beginning of the Section “Results.”

#### Measures

##### Primary measures

The D/N and HTT tasks are well-established measures of executive function, widely used in developmental research and suitable for children as young as 3 years of age (Gerstadt et al., 1994; Ponitz et al., 2009). For D/N, children are presented with two kinds of cards; either a white card depicting a yellow sun (day card) or a black card depicting a white moon and stars (night card). They are instructed to play a “silly” game in which they must say “day” when they see a night card and “night” when they see a day card. Hence, they must inhibit the pre-potent response to say the word that is associated with the picture, and say the opposite word. Children receive 16 cards presented in one of two predetermined pseudorandom orders. They are allowed to self-correct after an initial response, before the next card is presented. Only the last response is scored. One point was awarded for each correct trial, for a maximum score of 16. For HTT, children are instructed to touch their toes when told “touch your head,” or to touch their head when told “touch your toes.” Children receive four practice trials followed by 10 test trials, comprising a mix of “head” and “toes” instructions given in 1 of 2 pre-determined random orders. Conventional scoring awards 2 points for a correct response on the first attempt, 1 point for a correct response on the last attempt (i.e., for self-correcting before the next command was given) and 0 points for an incorrect response, for a maximum score of 20 (HTT20). To bring the data for HTT more in line with the data for D/N, we also scored HTT awarding 1 point for a correct response (whether on the first attempt or after self-correction) and 0 points for an incorrect response, for a maximum score out of 10 (HTT10). We report the results for both methods of scoring the HTT task.

The SDQ (Goodman, 1997, 1999) is a widely used screening measure for parents and teachers of children aged 3–16 years. We used the American preschool version (for ages 3–4 years) for parents (SDQ-P) and the analogous version for teachers (SDQ-T). Respondents indicate the extent to which each of 25 attributes, some positive (e.g., Has at least one good friend”) and some negative (e.g., “Often loses temper), applies to the child on a 3-point likert scale (not true, somewhat true, certainly true). The 25 attributes are divided equally between 5 subscales; emotional symptoms, conduct problems, hyperactivity/inattention, peer problems and pro-social behavior. Scores for each subscale range from 0 to 10 and scores on the first four scales are summed to form a total difficulties score out of 40, with higher scores indicating greater challenges^11^. The hyperactivity/inattention subscale (which we used as a moderator in our analyses) includes items such as “Restless, overactive, cannot stay still for long” and “Constantly fidgeting or squirming.”

The SCBE-30 (LaFreniere and Dumas, 1995) comprises a list of 30 behaviors; 10 positive (e.g., “cooperates with other children) and 20 negative (e.g., “hits, bites or kicks other children,” “inactive, watches other children play”). Teachers indicate the frequency of observing each behavior on a 6-point likert scale (1 = never, 2 or 3 = sometimes, 4 or 5 = often, 6 = always). The SCBE-30 yields three subscales – social competence, anger/aggression, and anxiety/withdrawal. Scores for each sub scale represent the mean of the teacher’s ratings on the 10 items that contribute to the scale. Scores for the positive items – those that contribute to the social competence subscale – are reversed such that, for all three subscales, higher scores indicate greater challenges.

The PPVT-4 (Dunn and Dunn, 1997) is a standardized vocabulary measure with excellent reliability and validity and is widely used in the developmental literature. Children are presented with a matrix of four pictures and required to point to the picture that corresponds with a word the experimenter said aloud. We used the PPVT-4 as a moderator in our analyses. Administration followed standardized instructions.

##### Additional measures

The EVT-4 (Williams, 2007) is also standardized, with excellent reliability and validity and widely used. Children are presented with a picture (e.g., a key), the experimenter poses a prompting question (e.g., “What is this?”) and children respond verbally. Administration followed standardized instructions.

The GRTR (Whitehurst and Lonigan, 2001) is a screening tool that assesses progress in developing early literacy skills in preschoolers. Children are presented with a matrix of four items (symbols, text, or pictures) and asked to indicate, e.g., which picture contains letters or which letter corresponds to a particular sound. Performance on the GRTR is significantly correlated with other measures of language and letter knowledge (Whitehurst and Lonigan, 2001). Children are awarded 1 point for each correct response for each of twenty-two trials to yield a maximum score of 22.

PTX (Wynn, 1992) taps children’s understanding of counting principles (one-to-one correspondence, stable order and cardinality), essential to helping get mathematical skills off the ground. Participants were presented with two arrays of black squares simultaneously, and required to point to the array with the number of squares that corresponds to a number the experimenter said aloud. The quantity of squares in the arrays ranged from 1 to 9. When the quantity depicted exceeds three (the majority of the trials) children cannot simply subitize (know by looking) and must count the squares in each array to be able to respond correctly. Children received 1 point for each correct response on each of 16 trials for a maximum score of 16.

Finally, the EDI (Janus and Offord, 2007) is a developmental checklist completed by teachers to assess overall development in young children. It was designed as a community or population measure rather than for individual diagnosis or screening. Researchers submit their raw data to the developers for the derivation of summary scores that are shared with the investigators and added to a central database to further enhance neighborhood, regional and national representation. The EDI has good psychometric properties, has been used in research internationally to inform regional and national policy on early childhood care and education^12^. The checklist comprises 103 items that probe observable behavior and competencies in 5 domains; physical well-being, language and cognitive development, social competence, emotional maturity, communication and general knowledge. Example items for each scale, respectively, include; “proficiency at holding a pen, crayon or brush,” “is able to attach sounds to letters” and “remembers things easily,” “is able to play with various children,” “is nervous, high-strung or stressed,” “ability to tell a story,” and “answers questions about the world.” Teachers rate the child in question on each item on Likert scales that vary across the instrument sections. Higher scale scores indicate greater maturity.

##### Procedures

Children were tested individually, in a quiet location in their preschool by a trained experimenter during regular preschool hours. The battery of measures was divided into two test sessions of about 30 min each. To help maintain motivation, each session included a variety of measures and breaks were given as needed. Parents and teachers completed their assigned questionnaires on a schedule to roughly coincide with the student data collection. The same teacher completed the teacher questionnaires at all data collection time points.

## Results

### Overview

We first report the results for Tools implementation fidelity, essential for interpretation of the results for the student outcomes. We then report the results from analyses of the student outcomes addressing each of our three research questions in turn: (1) Was there a main effect of curriculum? (2) Was there a main effect of curriculum moderated by initial language skills? and (3) Was there a main effect of curriculum moderated by initial level of hyperactivity/inattention? For each question, we report the results from the analyses of the data for Cohort A only, followed by the results from the analyses for the data from Cohorts A and B combined.

### Tools Implementation Fidelity

We derived inter-rater reliability by adding the number of elements that the observers agreed were either present (both said yes) or absent (both said no) and then converting the total to a percentage of 119 (the number of possible observable core elements) at each time point, for each site. We focused on the elements of the core activities because they were essential for the Tools program to be considered “in place.” We then calculated the mean percentage of inter-rater agreement for sites in the two groups, at F1, F2, and F3. Occasionally one or both observers were unable to observe an activity or to visit a site on the designated day (e.g., due to transit disruption in severe weather). Activities captured by only one observer were omitted from inter-rater analyses, when both observers missed activities, inter-rater agreement was based on the remaining observed activities, and in the rare case of a missed site, mean inter-rater agreement was based only on the sites attended. We used the same procedure to derive inter-rater reliability on the elements associated with the additional activities at F2 and F3 and report these results separately.

In general, inter-rater reliability for the 119 core elements was very high; at F1 it was 99.7% (range 99.2–100) and 97.4% (range 94.5–99.3); at F2, it was 98.4% (range 97.7–98.8) and 93% (range 81.2–98.9); and at F3, it was 87.7% (range 79.7–95.3) and 83% (range 75.6–94.3), for the YMCA PTL and Tools groups, respectively. For the 35 additional elements assessed at T2, inter-rater reliability was 99.6% (range 97.8–100) and 93.12% (range 86.7–98.2), and for the 41 additional elements assessed at T3, it was 100 and 92.7% (range 87.1–97.8), for the YMCA PTL and Tools groups, respectively. The somewhat higher (and less variable) agreement in the YMCA PTL group compared to the Tools group reflects the fact that the observers simply had to agree that the Tools activity (and therefore all of its elements) never occurred.

We calculated implementation fidelity by tallying the number of elements that were present and converting the total to a percentage of the 119 core elements, adjusting for missed activities, at each site, and for each time point. An element was counted as present if both observers agreed that it took place. It was also counted as present on the rare occasion that only one observer was in attendance (see above) and indicated that an element occurred, because inter-rater agreement was very high (see the Section “Results”). We used the same procedure to derive the mean percentage of additional elements implemented at F2 and F3 and report these results separately.

**FIGURE 2:**
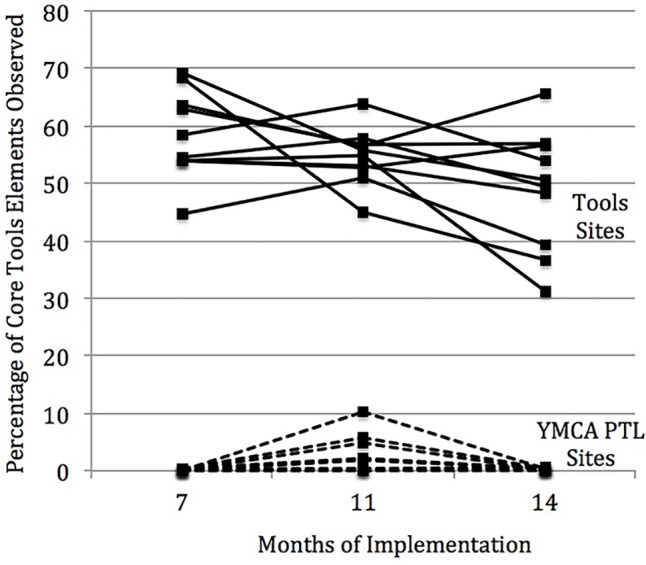
Implementation fidelity of the Tools curriculum. The percentage of core Tools elements observed at each time point are shown for individual sites. Only the teachers in the Tools group implemented Tools and they did so with moderate success. As expected with any new initiative, there was cross-site variability in the degree of Tools implementation fidelity.

**Figure 2** shows the results for implementation fidelity of the Tools program. The mean percentage of core elements implemented, based on the sites visited, was 0.15% (range 0–0.4) and 58.4% (range 44.8–69.2) at F1, 1.9% (range 0–5.8) and 54.7% (range 44.9–63.7) at F2, and 0.35% (range 0–0.8) and 48.9% (range 31.3–65.6) at F3, for YMCA PTL and Tools, respectively. As the figure reveals, the type of instruction occurring in the two groups was clearly different. Whereas the Tools activities were virtually absent from the YMCA PTL classrooms, teachers were moderately successful at implementing them in the Tools classrooms. As expected for implementing a new program, there was also considerable cross-site variability in Tools implementation fidelity.

The mean percentage of additional elements implemented was 0 and 0.6% (range 0–2.8) at F2 and 0 and 11.48% (range 0–37.83) at F3, for YMCA PTL and Tools, respectively. As expected, the additional activities were completely absent from the YMCA PTL group. For the Tools group, we found virtually no evidence of implementation at F2 and only modest evidence of implementation of the additional elements at F3, again with cross-site variability.

### Analysis of Student Outcomes

#### Method of Analysis

In all main analyses, we used a multilevel model to deal with the nesting of the data: children (level-1) who are nested within site (level-2). Multilevel analyses have been deemed ideal to investigate the effects of randomized controlled trials (Wears, 2002). Because the number of sites was relatively low (*n* = 20), we used Bayesian estimation which has been shown to have adequate performance under this condition (Hox et al., 2012). This estimation method allowed us to include participants with missing data, including those lost to attrition, based on the missing-at-random assumption (Asparouhov and Muthén, 2010). Accordingly, and because we omit treatment fidelity here, these analyses may be interpreted as intent-to-treat (Gupta, 2011). With Bayesian estimation, significance is assessed based on the 95% credible interval (95% CI). A parameter is significant if the 95% CI does not include zero (i.e., it is significantly different from zero).

We ran three sets of analyses to address our three research questions. First, we tested for a main effect of curriculum on child outcomes by looking at the effect of curriculum on the average level for child outcomes at the site-level (i.e., at level-2 because randomization occurred at the site-level) while controlling for initial levels of the outcome being tested (i.e., T1 score for each child). The following covariates were included: child sex, child age at the final assessment, initial levels of the outcome being tested (level-1) and curriculum assignment (level-2).^13^ Second, we asked whether the curriculum effect varied as a function of children’s initial language ability. We did this by adding an interaction term at level-1 between the curriculum assignment and language at T1. And third, we asked whether the curriculum effect varied as a function of children’s initial level of hyperactivity/inattention. Accordingly, we included an interaction term at level-1 between the curriculum assignment and parent ratings of hyperactivity/inattention at T1.

#### Main Effect of Curriculum

The results from the analyses of the main effect of curriculum are shown in **Table 3**. Specifically, we tested whether there were differences in the average level of children’s outcome in each site as a function of curriculum assignment. Because there were a large number of analyses, we only report the main effect of curriculum on each outcome for each analysis. We found no evidence that curriculum had an effect on the primary outcomes, either for cohort A only (see **Table 3A**) or for cohorts A and B combined (see **Table 3B**). That is, there were no differences on any of the primary outcomes between children in sites who received Tools and YMCA PTL instruction.

**Table 3:** Results for main effect of curriculum.

	Unstandardized	95% CI
	estimate	
**(A) Cohort A only**		
Day/night	0.17	[–0.787, 1.160]
Head to toes score out of 10	0.143	[–1.007, 1.276]
Head to toes score out of 20	–0.964	[–2.544, 0.567]
SDQ-parent total difficulties	0.526	[–1.976, 2.967]
SDQ-teacher total difficulties	–0.919	[–4.602, 2.930]
SCBE-30 anxiety/withdrawal	0.085	[–0.261, 0.449]
SCBE-30 anger/aggression	0.083	[–0.296, 0.492]
SCBE-30 social competence	0.12	[–0.184, 0.449]
**(B) Cohorts A and B combined**		
Day/night	0.144	[–0.626, 0.880]
Head to toes score out of 10	0.426	[–1.039, 1.717]
Head to toes score out of 20	0.005	[–3.069, 2.808]
SDQ-parent total difficulties	0.326	[–1.802, 2.381]
SDQ-teacher total difficulties	0.180	[–2.542, 3.046]
SCBE-30 anxiety/withdrawal	0.077	[–0.207, 0.380]
SCBE-30 anger/aggression	0.046	[–0.274, 0.398]
SCBE-30 social competence	0.117	[–0.222, 0.489]

*Models control for baseline score on each outcome, child sex and child age at T3 [when the outcome was measured). The effect was not significant for any of the outcome measures.*

#### Effect of Curriculum Moderated by Initial Language

The results from the analyses addressing whether or not there was an effect of curriculum moderated by initial language ability are shown in **Table 4**. Specifically, we tested whether or not the effect of curriculum varied systematically as a function of a child’s initial language ability, i.e., a significant curriculum by T1 language ability interaction. We report only the estimate pertaining to the interaction term, which was not significant, either for Cohort A only (**Table 4A**) or when the two cohorts were combined (**Table 4B**). Thus, we found no evidence that the effect of the Tools curriculum varied as a function of children’s initial language ability.

**Table 4:** Results for effect of curriculum moderated by initial language ability.

	Unstandardized	95% CI
	estimate	
**(A) Cohort A only**		
Day/night	0.157	[–0.456, 0.799]
Head to toes score out of 10	0.256	[–0.717, 1.218]
Head to toes score out of 20	0.302	[–1.717, 2.298]
SDQ-parent total difficulties	0.1O0	[–1.776, 1.963]
SDQ-teacher total difficulties	0.226	[–2.250, 2.729]
SCBE-30 anxiety/withdrawal	0.021	[–0.182, 0.223]
SCBE-30 anger/aggression	0.141	[–0.162, 0.444]
SCBE-30 social competence	–0.084	[–0.337, 0.169]
**(B) Cohorts A and B combined**		
Day/night	0.081	[–0.522, 0.670]
Head to toes score out of 10	0.361	[–1.035, 1.748]
Head to toes score out of 20	0.651	[–1.908, 3.200]
SDQ-parent total difficulties	0.275	[–1.554, 2.093]
SDQ-teacher total difficulties	0.154	[–1.924, 2.289]
SCBE-30 anxiety/withdrawal	–0.043	[–0.244, 0.174]
SCBE-30 anger/aggression	0.121	[–0.139, 0.377]
SCBE-30 social competence	–0.041	[–0.290, 0.212]

*Unstandardized estimates pertain to the interaction term only. Model controls for baseline score on each outcome, child sex, child age at T3 (when the outcome was measured) and children’s initial language ability (standardized PPVT score). The effect was not significant for any of the outcome measures.*

#### Effect of Curriculum Moderated by Initial Hyperactivity/Inattention

Finally, the results from the analyses addressing whether or not there was an effect of curriculum moderated by initial levels of hyperactivity/inattention are shown in **Table 5**. Specifically, we tested whether or not the effect of curriculum varied systematically as a function of initial level of hyperactivity/inattention, i.e., a significant curriculum by T1 level of hyperactivity/inattention (as indicated on the SDQ-P) interaction. The pattern of results was the same for cohort A (**Table 5A**) as for cohorts A and B combined (**Table 5B**), although the results were somewhat stronger for the larger combined sample.

**Table 5:** Results for effect of curriculum moderated by initial level of hyperactivity/inattention.

	Unstandardized	95% CI
	estimate	
**(A) Cohort A only**		
Day/night	0.188	[–0.393, 0.781]
Head to toes score out of 10	1.030^∗^	[0.215, 1.868]
Head to toes score out of 20	1.933^∗^	[0.275, 3.613]
SDQ-parent total difficulties	0.245	[–1.639, 2.124]
SDQ-teacher total difficulties	1.023	[–1.439, 3.410]
SCBE-30 anxiety/withdrawal	0.053	[–0.143, 0.240]
SCBE-30 anger/aggression	0.23	[–0.058, 0.514]
SCBE-30 social competence	0.076	[–0.176, 0.324]
**(B) Cohorts A and B combined**		
Day/night	0.253	[–0.305, 0.829]
Head to toes score out of 10	1.490^∗^	[0.264, 2.550]
Head to toes score out of 20	2.296^∗^	[0.159, 4.242]
SDQ-parent total difficulties	0.123	[–1.731, 1.913]
SDQ-teacher total difficulties	0.562	[–1.485, 2.629]
SCBE-30 anxiety/withdrawal	0.096	[–0.109, 0.282]
SCBE-30 anger/aggression	0.199	[–0.051, 0.443]
SCBE-30 social competence	0.048	[–0.198, 0.300]

*^∗^*p* < 0.05. Unstandardized estimates pertain to the interaction term only. Model controls for baseline score on each outcome, child sex, child age at T3 (when the outcome was measured) and initial parent rating of hyperactivity/inattention.*

There were significant interactions for HTT10 (i.e., when scoring only as correct or incorrect) and HTT20 (i.e., using the conventional scoring system for HTT) only. To interpret these findings, we report the standardized parameter estimate as a measure of the effect size of curriculum at different levels of child hyperactivity/inattention (1 SD below average, average, 1 SD above average).

For HTT10, the effect of curriculum was significant at trend level for cohort A and significant at the conventional level for cohorts A and B combined, for children with above average, but not average or below average hyperactivity/inattention. The values for cohort A were β = 0.308, 95% CI [-0.018, 0.641] and 90% CI [0.037, 0.582], β = 0.063, 95% CI [-0.190, 0.319] and β = -0.179, 95% CI [-0.499, 0.127], and the values for the combined cohorts were β = 0.483, 95% CI [0.068, 0.846], β = 0.141, 95% CI [-0.146, 0.395] and β = -0.202, 95% CI [-0.570, 0.166], for above average, average and below average hyperactivity/inattention, respectively.

For HTT20, the effect of curriculum was not significant for any of the hyperactivity subgroups, either for cohort A or for the two cohorts combined. The values for cohort A were β = 0.250, 95% CI [-0.153, 0.662], β = -0.007, 95% CI [-0.345, 0.328] and β = -0.262, 95% CI [-0.650, 0.128], and the values for the combined cohorts were β = 0.338, 95% CI [-0.115, 0.755], β = 0039, 95% CI [-0.293, 0.355] and β = -0.261, 95% CI [-0.655, 0.158], for above average, average and below average hyperactivity/inattention, respectively.

Thus, we found that amongst children with high levels of initial hyperactivity/inattention, those who received Tools instruction showed significantly greater improvement on our behavioral measure of executive function, one of our key outcome measures, than their peers who received YMCA PTL instruction.

## Discussion

We investigated the effectiveness of the Tools of the Mind preschool curriculum for improving self-regulation in a diverse sample of Canadian preschoolers. We were primarily interested in whether or not Tools instruction would lead to greater improvement on measures of self-regulation and executive function compared to YMCA PTL instruction (the business-as-usual approach) and in whether the effects of curriculum might be moderated by children’s initial language and hyperactivity/inattention.

We did not find a main effect of curriculum or a significant interaction between curriculum and children’s initial language skills, on any of our outcome measures. However, we found a significant interaction between curriculum and children’s initial level of hyperactivity/inattention on one of our executive functions tasks. Amongst children with high levels of hyperactivity/inattention, those who received Tools instruction showed significantly greater improvement than those who received YMCA PTL instruction on HTT. The interaction was not significant for D/N, or for any of the scales derived from parents and teachers responses on the questionnaires. In keeping with some previous research then, we found a benefit of Tools instruction for children experiencing the greatest challenges in the development of self-regulation (Diamond et al., 2007; Barnett et al., 2008; Blair and Raver, 2014).

The absence of a main effect of curriculum in the present study contributes to the mixed results from five previous studies evaluating the impact of the full Tools curriculum; three of which found positive effects of Tools and two of which reported positive effects of the comparison curricula. Notably, two of the studies with positive effects of Tools were conducted with homogeneous, low SES, preschool samples (Diamond et al., 2007; Barnett et al., 2008), and in the third study with a larger more variable kindergarten sample, the effects of Tools were considerably stronger in high poverty schools (Blair and Raver, 2014). Our sample of preschoolers varied considerably in SES, perhaps obscuring the benefit of Tools to the low SES children (we were not able to test for SES effects directly as these data were not available for individual children). At least one of the other studies using the full Tools curriculum also had a large, low SES sample (Farran and Wilson, 2014; SES details for the study by Lonigan and Phillips, 2012, which is unpublished, were not available), but the lack of positive effects of Tools in that study could be due to specific features of the comparison group instruction which differed from the comparison curricula in the preschool studies that found positive effects of Tools (see **Table 1**).

The lack of a significant interaction between initial language and curriculum in the present study was somewhat surprising because language plays such a central role in the Tools curriculum. In Tools, language is not only important for interpersonal communication, but many activities in the Tools daily routine effectively instruct children, and provide plenty of practice, in using language to regulate their behavior. One would therefore expect that boosting these capacities in children with relatively poor language skills might lead to greater gains in self-regulation. On the other hand, that we did not find a significant interaction between curriculum and initial language skills indicates that both types of instruction are suitable for children at all levels of language development (i.e., provided they have sufficient capacity to understand the teacher’s instructions, which was a criterion for participation in the present study).

However, we found a significant interaction between initial hyperactivity/inattention and curriculum indicating significantly greater gains from Tools for children with high hyperactivity/inattention. It may be crucial for these children to have numerous, routinized opportunities, distributed throughout the day to practice self-regulation because their high level of activity and inattentiveness may impede their ability to profit from the opportunities to practice self-regulation that are par for the course of the typical preschool day. That the Tools curriculum is built around a set of self-regulating activities may increase the likelihood that struggling children will have more frequent self-regulating experiences and reap the associated rewards.

It is noteworthy that the interaction between curriculum and initial level of hyperactivity/inattention was significant at trend level for cohort A and significant at the conventional alpha level for the two cohorts combined (likely due to greater statistical power to detect significant effects). The consistent pattern of findings suggests that the benefit of Tools was already beginning to take hold in cohort B, who entered the study later and thus had a shorter duration of exposure to Tools. These children may have benefitted from entering a classroom where the Tools program was already underway. This is in line with the Tools theoretical framework that emphasizes the social context of learning. Moreover, the finding that high hyperactive/inattentive children benefited more from Tools, but that children with hyperactivity/inattention in the average range fared similarly well in the two programs shows that Tools can help students who struggle with self-regulation at no apparent cost to those who are less challenged in this regard. These findings are useful for educators in daycare settings where enrollment is ongoing and who may be concerned about integrating new students struggling with self-regulation and the impact on other students.

It is interesting that although the interaction between curriculum and hyperactivity/inattention held for HTT whether we scored children’s response as right or wrong or we gave more credit for responding correctly on the first attempt (the conventional scoring method), the greater impact of Tools for high hyperactivity/inattention children only held for the first approach to scoring. It is possible that another approach to subgrouping children’s hyperactivity/inattention scores based on the conventional scoring method might yield greater insights regarding the children for whom Tools is most effective, which may have scientific merit regarding nuances in the development of self-regulation. However, for present purposes, the results for the abbreviated scoring system may be more meaningful. In the interests of improving behavior, and ultimately school readiness, the critical issue was if Tools instruction could bring about an improvement in children’s ability to rein themselves in at all.

It is also interesting that the observed benefits to the Tools children held for HTT but not for D/N, tasks with highly similar cognitive demands but different response modalities. Highly active or inattentive children may enjoy HTT more as it capitalizes on their propensity to move and may find it more challenging to sit still and attend to the cognitive demands of D/N. Young children may also be more familiar with action based inhibitory control games like Simon says and musical chairs that are similar to HTT. Finally, the fewer test trials for HTT (10) vs. D/N (16) may also reduce task demands enough for struggling children to demonstrate the extent of their development in self-regulation. It is possible that Tools instruction may eventually yield sufficient improvement in behavioral regulation for better performance on D/N, and these improvements may eventually translate to better behavior at home and at school. In other words, HTT may be optimally suited to tap early, subtle improvements in self-regulation in high hyperactivity/inattention children that were only beginning to occur.

A strength of the present study is that we compared the effectiveness of Tools, a program that targets pretend play, language, and self-regulation, to YMCA PTL another preschool program that promotes quality play but does not target self-regulation specifically. The benefit of Tools to growth in executive function, at least to some children, suggests that the Tools self-regulatory activities may be an important ingredient of the program. In other words, Tools instruction may benefit learners through its emphasis on self-regulation over and above its emphasis on quality play. Indeed, another reason for the greater sensitivity of HTT compared to D/N for revealing improvements in high hyperactivity/inattentive children may be because Tools actually includes activities that are highly similar to HTT (like the freeze game).

Other strengths include random assignment at the site level to the two curricula at the study outset; standardized resources, overall quality of care and teacher preparation across all sites in the participating organization; and that both groups of teachers received professional development and ongoing coaching support in their respective curricula to help sustain acceptable quality in program delivery. Implementation fidelity data confirmed that only the Tools teachers implemented the Tools curriculum and that they did so with moderate success.

The present research also faced challenges beyond our control that could have impacted the study outcome. The participating sample was somewhat limited, perhaps constraining our power to detect further significant effects if present. That said, it was comparable to that in two similar previous studies that also reported significant effects of Tools on selected outcomes (Diamond et al., 2007; Barnett et al., 2008), one of which also included two cohorts of children entering the study at different times (Diamond et al., 2007). On the other hand, studies with larger samples have failed to turn up any significant effects of Tools (see Lonigan and Phillips, 2012; Farran and Wilson, 2014).

Other factors may have posed challenges to the Tools teachers’ ability to reach and to sustain a high level of program fidelity. To be sure, teachers efforts to implement Tools was likely affected by the attrition that occurred just 5 months after the study got underway and the influx of a new cohort of younger children. The disruption likely posed greater challenges for the Tools teachers’ who faced a considerable adjustment in pedagogical mindset. This notion is supported by the fact that implementation fidelity in the Tools classrooms was only moderately high, even with ongoing coaching support to help teachers stay on track. Another consideration is that since we did not assess implementation fidelity of the YMCA PTL program, it remains possible that teachers in the Tools group continued to implement some aspects of YMCA PTL, in which they were fully trained prior to participating in the study. However, we believe that this is unlikely. The Tools daily schedule comprises a set of prescribed activities that leave little room for other practices. Indeed, further inspection of the fidelity data showed that the moderate level of success reflected missing elements of the core Tools activities rather than neglecting to implement those activities at all, which may also be a consequence of adapting to the considerable change that occurred to the class composition. Thus, it remains possible that without serious disruptions to attendance, Tools teachers may be able to sustain a sufficiently high level of program fidelity for further gains to manifest in observable behavior.

The focus in previous studies of Tools effectiveness on low SES samples is understandable in light of the well-established link between poverty and school readiness (see e.g., Duncan et al., 1994). However, many preschools serve diverse populations. Moreover, Moffitt et al. (2011) have shown that low self-control is related to poorer outcomes in adulthood – after controlling for SES – and that improving self-control between childhood and adolescence can lead to better outcomes in adulthood. Since SES disparities in school readiness appear to be mediated by children’s cognitive skills (e.g., Fitzpatrick et al., 2014), it may be more useful to early childhood educators to understand the cognitive and behavioral characteristics (such as language and hyperactivity/inattention) of the children for whom Tools is most effective, in determining the curriculum best suited to the populations they serve.

That both programs promote quality play and children in the two groups made similar gains on most of the study measures suggests that the Tools and YMCA PTL curricula both have merits for meeting the needs of children for whom self-regulation is in the typical range. However, Tools may offer an advantage in classrooms with children experiencing greater challenges to self-regulation. Importantly, our findings that Tools did not have a negative impact on children with hyperactivity/inattention in the average range suggests that the positive impact of Tools on children with high hyperactivity/inattention can occur without disadvantage to children less challenged in this regard. Given the considerable, long-term consequences of poor self-regulation and its impact on developing a healthy quality of life, the Tools program may be a worthy investment.

## Author Contributions

RT, BF, and PC conceptualized the study with input from LH. TS and RT designed the study. TS operationalized and executed it. AO, HF, PC, and GG contributed to data collection and coding. AP conceptualized and carried out the analytic plan with input from TS and RT. TS wrote the manuscript with input from all of the authors.

## Conflict of Interest Statement

The authors declare that the research was conducted in the absence of any commercial or financial relationships that could be construed as a potential conflict of interest.

## Footnotes

1 http://toolsofthemind.org/learn/what-is-tools/

2 http://toolsofthemind.org/about/history/

3 http://www.nytimes.com/2009/09/27/magazine/27tools-t.html?smid=pl-shareand_r=0

4 http://www.npr.org/templates/story/story.php?storyId=76838288

5 The YMCA was only able to provide fee subsidy data by site, not for individual families. Hence, we could not include this proxy for SES in our analyses. However, analyzing the data in terms of the impact of Tools as a function of initial language and hyperactivity/inattention allowed us to go beyond simply replicating earlier findings regarding SES to better understand why Tools might sometimes work for low SES children.

6 Children attended the daycare year-round, typically with a short break in the summertime for family vacation.

7 Ethnicity data are not typically collected as part of daycare enrollment in Canada and we were not able to collect such data systematically as part of the study. Nor could we appeal to census data by daycare postal code as researchers sometimes do because some of our participating sites served commuter populations. However, the sites spanned the geographical limits of a large cosmopolitan city and our research team observed that the sample varied widely in ethnic composition.

8 See Supplementary Table S1 in the supplementary online materials for baseline data (collected at T1) for the participants in cohort A who withdrew and for those who remained in the study by T2.

9 See Supplementary Table S2 in the supplementary online materials for performance on the various measures for cohort A children who remained in the study and for cohort B children entering the study, at T2.

10 www.toolsofthemind.org

11 www.sdqinfo.com

12 https://edi.offordcentre.com/resources/bibliography-of-the-edi

13 There were no significant effects of sex in any of the models with D/N or HTT as outcome variables. See Supplementary Table S3 in the supplementary online materials.

## References

[B1] Asparouhov T. Muthén B. (2010). *Bayesian Analysis Using Mplus: Technical Implementation (Version 3). Technical Report.* Available at: http://statmodel.com/papers.shtml

[B2] Assel M. Landry S. H. Swank P. R. Gunnewig S. (2007). An evaluation of curriculum, setting, and mentoring on the performance of children enrolled in pre-kindergarten. *Read. Writ.* 20 463–494. 10.1007/s11145-006-9039-5

[B3] Barnett W. S. Jung K. Yaroz D. J. Thomas J. Hornbeck A. Stechuk R. (2008). Educational effects of the tools of the mind curriculum: a randomized trial. *Early Child. Res. Q.* 23 299–313. 10.1016/j.ecresq.2008.03.001 20846442

[B4] Blair C. Raver C. C. (2014). Closing the achievement gap through modification of neurocognitive and neuroendocrine function: results from a cluster randomized controlled trial of an innovative approach to the education of children in kindergarten. *PLOS ONE* 9:e112393. 10.1371/journal.pone.0112393 25389751 PMC4229187

[B5] Blair C. Razza R. P. (2007). Relating effortful control, executive function, and false belief understanding to emerging math and literacy ability in kindergarten. *Child Dev.* 78 647–663. 10.1111/j.1467-8624.2007.01019.x 17381795

[B6] Blair C. Ursache A. (2011). “A bidirectional theory of executive functions and self-regulation,” in *Handbook of Self-Regulation* 2nd Edn eds Vohs K. Baumeister R. (New York, NY: Guilford Press) 300–320.

[B7] Blakey E. Carroll D. J. (2015). A short executive function training program improves preschoolers working memory. *Front. Psychol.* 6:1827. 10.3389/fpsyg.2015.01827 26635710 PMC4656848

[B8] Bodrova E. Leong D. (2007). *Tools of the Mind* 2nd Edn. Englewood, CO: Prentice-Hall.

[B9] Bull R. Scerif G. (2001). Executive functioning as a predictor of children’s mathematics ability: inhibition, switching and working memory. *Dev. Neuropsychol.* 19 273–293. 10.1207/S15326942DN1903_3 11758669

[B10] Carlson S. M. (2005). Developmentally sensitive measures of executive function in preschool children. *Dev. Neuropsychol.* 28 595–616. 10.1207/s15326942dn2802_3 16144429

[B11] Clements D. Sarama J. Unlu F. Layzer C. (2012). The efficacy of an intervention synthesizing scaffolding designed to promote self-regulation with an early mathematics curriculum: effects on executive function. *Paper Presented at the Society for Research on Educational Effectiveness Conference* Washington, DC.

[B12] Diamond A. Barnett W. S. Thomas J. Munro S. (2007). Preschool program improves cognitive control. *Science* 318 1387–1388. 10.1126/science.1151148 18048670 PMC2174918

[B13] Diamond A. Lee K. (2011). Interventions shown to aid executive function development in children 4 to 12 years old. *Science* 333 959–964. 10.1126/science.1204529 21852486 PMC3159917

[B14] Domitrovich C. E. Cortes R. C. Greenberg M. T. (2007). Improving young children’s social and emotional competence: a randomized trial of the preschool “PATHS” curriculum. *J. Prim. Prev.* 28 67–91. 10.1007/s10935-007-0081-0 17265130

[B15] Duncan G. J. Brooks-Gunn J. Klebanov P. K. (1994). Economic deprivation and early childhood development. *Child Dev.* 65 296–318. 10.2307/1131385 7516849

[B16] Dunn L. M. Dunn L. M. (1997). *Peabody Picture Vocabulary Test* 3rd Edn. Circle Pines, MN: American Guidance Service.

[B17] Eden S. Huggins L. (2001). *Playing to Learn: A Guide to High Quality Education for Children from Infancy to Age Six* 1st Edn. Toronto, ON: YMCA.

[B18] Farran D. Wilson S. J. Lipsey M. Turner K. (2013). Effects through kindergarten of a prekindergarten curricular attempt to improve self-regulation and achievement. *Paper Presented at the Society for Research on Educational Effectiveness Conference* Washington, DC.

[B19] Farran D. C. Wilson S. J. (2014). *Achievement and Self-Regulation in Prekindergarten Classrooms: Effects of the Tools of the Mind Curriculum.* Nashville, TN: Peabody Research Institute Available at: https://my.vanderbilt.edu/toolsofthemindevaluation/files/2011/12/Tools-Submission-Child-Development-7-27-14.pdf

[B20] Fitzpatrick C. McKinnon R. D. Blair C. B. Willoughby M. T. (2014). Do preschool executive function skills explain the school readiness gap between advantaged and disadvantaged children? *Learn. Instr.* 30 25–31. 10.1016/j.learninstruc.2013.11.003

[B21] Foulon S. Pingault J. B. Larroque B. Melchior M. Falissard B. Côté S. M. (2015). Developmental predictors of inattention-hyperactivity from pregnancy to early childhood. *PLOS ONE* 10:e0125996. 10.1371/journal.pone.0125996 25938453 PMC4418828

[B22] Gerstadt C. L. Hong Y. J. Diamond A. (1994). The relationship between cognition and action: performance of 3.5 – 7 years old on a stroop-like day-night test. *Cognition* 53 129–153. 10.1016/0010-0277(94)90068-X 7805351

[B23] Goodman R. (1997). The strengths and difficulties questionnaire: a research note. *J. Child Psychol. Psychiatry* 38 581–586. 10.1111/j.1469-7610.1997.tb01545.x 9255702

[B24] Goodman R. (1999). The extended version of the strengths and difficulties questionnaire as a guide to child psychiatric caseness and consequent burden. *J. Child Psychol. Psychiatry* 40 791–801. 10.1111/1469-7610.00494 10433412

[B25] Gormley W. T. Jr. Gayer T. Phillips D. Dawson B. (2005). The effects of universal pre-k on cognitive development. *Dev. Psychol.* 41 872–884. 10.1037/0012-1649.41.6.872 16351334

[B26] Gupta S. K. (2011). Intention-to-treat concept: a review. *Perspect. Clin. Res.* 2 109–112. 10.4103/2229-3485.83221 21897887 PMC3159210

[B27] Hofmann W. Schmeichel B. J. Baddeley A. D. (2012). Executive functions and self-regulation. *Trends Cogn. Sci.* 16 174–180. 10.1016/j.tics.2012.01.006 22336729

[B28] Hox J. van de Schoot R. Matthijsse S. (2012). How few countries will do? Comparative survey analysis from a Bayesian perspective. *Surv. Res. Methods* 6 87–93. 10.18148/srm/2012.v6i2.5033

[B29] Janus M. Offord D. R. (2007). Development and psychometric properties of the early development instrument (EDI): a measure of children’s school readiness. *Can. J. Behav. Sci.* 39 1–22. 10.1037/cjbs2007001

[B30] Kena G. Aud S. Johnson F. Wang X. Zhang J. Rathbun A. (2014). *The Condition of Education 2014 (NCES 2014-083).* Washington, DC: U.S. Department of Education.

[B31] LaFreniere P. J. Dumas J. E. (1995). *Social Competence and Behavior Evaluation.* Los Angeles, CA: Western Psychological Services.

[B32] Ling D. S. Wong C. D. Diamond A. (2016). Do children need reminders on the day-night task, or simply some way to prevent them from responding too quickly? *Cogn. Dev.* 37 67–72. 10.1016/j.cogdev.2015.10.003 26949287 PMC4776648

[B33] Lonigan C. J. Phillips B. M. (2012). Comparing skills-focused and self-regulation focused preschool curricula: impacts on academic and self-regulatory skills. *Paper Presented at the Society for Research on Educational Effectiveness Conference* Washington, DC.

[B34] Luria A. R. (1966). *Higher Cortical Functions in Man.* New York, NY: Basic Books.

[B35] Martin S. Huggins L. (2015). *Playing to Learn: A Guide to High Quality Education for Children from Infancy to Age Six* 2nd Edn. Toronto, ON: YMCA.

[B36] Moffitt T. E. Arsenault L. Belsky D. Dickson N. Hancox R. J. Harrington H. (2011). A gradient of childhood self-control predicts health, wealth, and public safety. *Proc. Natl. Acad. Sci. U.S.A.* 108 2693–2698. 10.1073/pnas.1010076108 21262822 PMC3041102

[B37] Morris P. Mattera S. K. Castells N. Bangser M. Bierman K. Raver C. (2014). *Impact Findings from the Head Start CARES Demonstration: National Evaluation of Three Approaches to Improving Preschoolers’ Social and Emotional Competence.* OPRE Report 2014-44 Washington, DC: U.S. Department of Health and Human Services.

[B38] Noble K. G. McCandliss B. D. Farah M. J. (2007). Socioeconomic gradients predict individual differences in neurocognitive abilities. *Dev. Sci.* 10 464–480. 10.1111/j.1467-7687.2007.00600.x 17552936

[B39] Noble K. G. Norman M. F. Farah M. J. (2005). Neurocognitive correlates of socioeconomic status in kindergarten children. *Dev. Sci.* 8 74–87. 10.1111/j.1467-7687.2005.00394.x 15647068

[B40] Pingault J. B. Tremblay R. E. Vitaro F. Carbonneau R. Genolini C. Falissard B. (2011). Childhood trajectories of inattention and hyperactivity and prediction of educational attainment in early adulthood: a 16-year longitudinal population-based study. *Am. J. Psychiatry* 168 1164–1170. 10.1176/appi.ajp.2011.10121732 21799065

[B41] Ponitz C. C. McClelland M. M. Matthews J. S. Morrison F. J. (2009). A structured observation of behavioral self-regulation and its contribution to kindergarten outcomes. *Dev. Psychol.* 3 605–619. 10.1037/a0015365 19413419

[B42] Rimm-Kaufman S. E. Pianta R. C. Cox M. J. (2000). Teachers’ judgments of problems in the transition to kindergarten. *Early Child. Res. Q.* 15 147–166. 10.1016/S0885-2006(00)00049-1

[B43] Vallotton C. Ayoub C. (2011). Use your words: the role of language in the development of toddlers’ self-regulation. *Early Child. Res. Q.* 26 169–181. 10.1016/j.ecresq.2010.09.002 21969766 PMC3184006

[B44] Vitaro F. Larose S. Tremblay R. E. (2005). Kindergarten disruptive behaviors, protective factors, and educational achievement by early adulthood. *J. Educ. Psychol.* 97 617–629. 10.1037/0022-0663.97.4.617

[B45] Vygotsky L. (1967). Play and its role in the mental development of the child. *Sov. Psychol.* 3 6–18. 10.2753/RPO1061-040505036

[B46] Vygotsky L. (1978). *Mind in Society: The Development of Higher Psychological Processes.* Cambridge, MA: Harvard University Press.

[B47] Wears R. L. (2002). Advanced statistics: statistical methods for analyzing cluster and cluster-randomized data. *Acad. Emerg. Med.* 9 330–341. 10.1111/j.1553-2712.2002.tb01332.x 11927463

[B48] Welsh J. A. Nix R. L. Blair C. Bierman K. L. Nelson K. E. (2010). The development of cognitive skills and gains in academic school readiness for children from low-income families. *J. Educ. Psychol.* 102 43–53. 10.1037/a0016738 20411025 PMC2856933

[B49] Whitehurst G. J. Lonigan C. J. (2001). *Get Ready to Read!* Columbus, OH: Pearson Early Learning.

[B50] Williams K. (2007). *Expressive Vocabulary Test* 2nd Edn. San Antonio, TX: Pearson.

[B51] Wilson S. J. Farran D. C. (2012). Experimental evaluation of the tools of the mind curriculum in society for research on educational effectiveness. *Paper Presented at the Society for Research on Educational Effectiveness Conference* Washington, DC.

[B52] Wynn K. (1992). Children’s acquisition of the number words and the counting system. *Cogn. Psychol.* 24 220–251. 10.1016/0010-0285(92)90008-P

[B53] Zelazo P. D. Carlson S. M. (2012). Hot and cool executive function in childhood and adolescence: development and plasticity. *Child Dev. Perspect.* 6 354–360. 10.1111/j.1750-8606.2012.00246.x

