# Supplementary material for: A Cluster Randomized-Controlled Trial of the Impact of the Tools of the Mind Curriculum on Self-Regulation in Canadian Preschoolers
Source: Front Psychol. 2018 Jan 17;8:2366. doi: 10.3389/fpsyg.2017.02366 (PMC5782823; doi:10.3389/fpsyg.2017.02366)
Supplement: Supplementary file 1 [file Table_1.pdf]

Table 1S. Baseline (T1) performance for cohort A children who left and those who remained in the study by T2.

| | **Leavers** n = 78 **Mean (sd)** | **Stayers** n = 117 **Mean (sd)** |
|---|---|---|
| **Primary Measures** | | |
| Day/Night | 9.91 (4.6) | 10.74 (4.8) |
| Head to Toes/20 | 4.65 (6.6) | 7.44 (7.6) |
| Head to Toes / 10 | 3.07 (3.8) | 4.21 (4.1) |
| SDQ-P Total Difficulties | 9.49 (5.0) | 8.40 (5.1) |
| SDQ-T Total Difficulties | 8.27 (5.0) | 6.53 (5.9) |
| SCBE-30 Anger/Aggression | 2.25 (.72) | 2.09 (.81) |
| SCBE-30 Anxiety/Withdrawal | 2.94 (.73) | 2.74 (.92) |
| SCBE-30 Social Competence | 2.20 (.68) | 2.02 (.64) |
| PPVT-4 Standard Score | 97.5 (12.7) | 103.4 (16.0) |
| **Additional Measures** | | |
| EVT-4 Standard Score | 102.7 (14.2) | 108.2 (16.7) |
| GRTR-R (reading) | 12.16 (4.7) | 12.36 (4.8) |
| PTX (math) | 10.8 (2.7) | 11.33 (2.5) |
| EDI-R Physical Well-Being | 8.9 (1.1) | 8.9 (1.0) |
| EDI-R Social Competence | 7.42 (1.8) | 7.47 (1.9) |
| EDI-R Emotional Maturity | 7.35 (1.4) | 7.45 (1.6) |
| EDI-R Language/Cognitive Development | 5.8 (2.0) | 5.8 (1.9) |
| EDI-R Communication/General Knowledge | 7.4 (2.5) | 7.8 (2.4) |

Independent-samples t-tests revealed no significant differences between group means, based on P<.02, the critical alpha level after Bonferroni adjustment for multiple comparisons. See methods in main body of paper for details regarding measures.
