# Supplementary material for: A Cluster Randomized-Controlled Trial of the Impact of the Tools of the Mind Curriculum on Self-Regulation in Canadian Preschoolers
Source: Front Psychol. 2018 Jan 17;8:2366. doi: 10.3389/fpsyg.2017.02366 (PMC5782823; doi:10.3389/fpsyg.2017.02366)
Supplement: Supplementary file 2 [file Table_2.pdf]

Table 2S. Performance at T2 by cohort.

	Cohort A<br>n = 117<br>Mean (sd)	Cohort B<br>n = 61<br>Mean (sd)
**Primary Measures**		
Day/Night	13.54 (3.7)	12.46 (4.1)
Head to Toes/20*	13.26 (7.6)	5.96 (7.0)
Head to Toes / 10*	7.10 (4.0)	3.45 (3.9)
SDQ-P Total Difficulties	8.27 (4.7)	7.95 (5.3)
SDQ-T Total Difficulties*	5.97 (5.6)	8.57 (5.4)
SCBE-30 Anger/Aggression	2.04 (1.29)	2.21 (.77)
SCBE-30 Anxiety/Withdrawal	1.83 (.64)	2.05 (.55)
SCBE-30 Social Competence*	2.49 (.91)	3.0 (.79)
PPVT-4 Standard Score	108.67 (13.4)	106.36 (15.0)
**Additional Measures**		
EVT-4 Standard Score	110.94 (14.7)	108.1 (17.4)
GRTR-R (reading)*	17.83 (4.8)	12.95 (4.47)
PTX (math)*	13.2 (2.0)	11.38 (3.16)
EDI-R Physical Well-Being	8.9 (1.2)	8.5 (1.04)
EDI-R Social Competence*	7.92 (1.8)	6.76 (1.9)
EDI-R Emotional Maturity*	7.88 (1.5)	7.02 (1.4)
EDI-R Language/Cognitive Development*	7.61 (1.9)	5.56 (2.0)
EDI-R Communication/General Knowledge*	8.28 (2.05)	6.39 (2.5)

* denotes measures with significant differences between group means based on P<.02, the critical alpha level after Bonferroni adjustment for multiple comparisons. Cohort A children were significantly older than their cohort B peers (P<.0001) and scored significantly better on several measures at T2. See methods section in main body of the paper for details regarding measures.
