# Supplementary material for: A Cluster Randomized-Controlled Trial of the Impact of the Tools of the Mind Curriculum on Self-Regulation in Canadian Preschoolers
Source: Front Psychol. 2018 Jan 17;8:2366. doi: 10.3389/fpsyg.2017.02366 (PMC5782823; doi:10.3389/fpsyg.2017.02366)
Supplement: Supplementary file 3 [file Table_3.PDF]

Table 3s. Effect of sex in the models predicting performance on the Day/Night and Head to Toes tasks

	Cohort A		Cohort A+B	
	Estimate	95% CI	Estimate	95% CI
		**Main effect of curriculum**		
Day/Night	-0.16	-0.70, 0.39	0.03	-0.41, 0.45
Head to Toes/10	0.63	-0.22, 1.50	0.56	-0.39, 1.52
Head to Toes/20	0.82	-0.59, 2.27	1.11	-0.54, 2.75
		**Moderated by language**		
Day/Night	-0.26	-0.82, 0.28	-0.06	-0.52, 0.39
Head to Toes/10	0.56	-0.27, 1.39	0.44	-0.42, 1.32
Head to Toes/20	1.23	-0.48, 1.06	1.06	-0.62, 2.75
		**Moderated by parent rated hyperactivity/inattention**		
Day/Night	-0.18	-0.72, 0.38	0.01	-0.42, 0.45
Head to Toes/10	0.73	-0.12, 1.56	0.66	-0.22, 1.53
Head to Toes/20	1.56	-0.05, 3.17	1.27	-0.32, 2.89

* $p < .05$, hence there were no significant effects of sex in any of the models with Day/Night and Head to Toes as outcome variables.
